# Factitious Cushing's Syndrome: A Diagnosis to Consider When Evaluating Hypercortisolism

**DOI:** 10.3389/fendo.2019.00129

**Published:** 2019-03-04

**Authors:** Maria M. Pineyro, Lia Redes, Sylvana De Mattos, Luciana Sanchez, Estefania Brignardello, Virginia Bianchi, Vanessa Ems, Dardo Centurión, Marcelo Viola

**Affiliations:** ^1^Clínica de Endocrinología y Metabolismo, Facultad de Medicina, Hospital de Clínicas, Universidad de la República, Montevideo, Uruguay; ^2^Clínica Psiquiátrica, Facultad de Medicina, Hospital de Clínicas, Universidad de la República, Montevideo, Uruguay; ^3^Departamento de Anatomía Patológica, Facultad de Medicina, Hospital de Clínicas, Universidad de la República, Montevideo, Uruguay; ^4^Clínica Quirúrgica 1, Facultad de Medicina, Hospital Pasteur, Universidad de la República, Montevideo, Uruguay

**Keywords:** hypercortisolism, Cushing's syndrome, factitious Cushing's syndrome, synthetic glucocorticoid screen, liquid chromatography-tandem mass spectrometry

## Abstract

Factitious Cushing's syndrome is exceptionally rare. The diagnosis is challenging due to the interference of exogenous corticosteroids with cortisol immunoassays. We present a case of a 26 year old female that presented with clinical and biochemical features of Cushing's syndrome. She denied any exogenous corticosteroid use. She had a suppressed ACTH level with normal adrenal glands on CT scans. There was a paradoxical increase of cortisol with a 100% rise in 24 h urinary free cortisol (UFC) during the Liddle's test suggestive of primary pigmented nodular adrenocortical disease (PPNAD). However, basal UFC levels were within normal values, interpreted as an intermittent variation of cortisol secretion maybe due to cyclic Cushing's. At this point a synthetic glucocorticoid serum screening was ordered, which was denied by the administrators because the test was not available in our hospital. A positron emission tomography (PET)-CT using 18 F-Flurodeoxyglucose did not show any uptake in the adrenal glands. With the diagnosis of probable primary pigmented nodular adrenocortical disease a unilateral right adrenelectomy was performed. Histopathological examination revealed normal adrenal gland. A synthetic glucocorticoid serum screen by liquid chromatography-tandem mass spectrometry (LC-MS/MS) sent to Mayo Clinic lab revealed high levels of serum prednisone and prednisolone. In conclusion, factitious Cushing's syndrome is an important diagnosis to consider in patients being evaluated for hypercortisolism. Discordant hormonal test results as well as normal findings on adrenal glands on CT scan should raise suspicion of this entity, and prompt measurement of synthetic corticosteroids using LC-MS/MS.

## Background

Factitious disorder imposed on self is a psychiatric disorder characterized by intentional fabrication of physical or physiological symptoms and/or signs, without an obvious gain (DSM-5). In contrast to malingering, who fabricate symptoms for obvious external reward such as financial gain, avoiding military duty or work, the motivation of patients with factitious disorder is to receive medical attention.

The surreptitious administration of several hormones has been reported, such as cathecholamine injection mimicking pheocromocytoma, insulin imitating insulinoma, and thyroid hormone ingestion feigning thyrotoxicosis (1–3). Exogenous ingestion of corticosteroids is the most common cause of Cushing's syndrome.

Reports of factitious Cushing's syndrome are extremely rare. In addition, the diagnosis is challenging due to interference of synthetic corticosteroids and their metabolites with cortisol immunoassays (4). We present a case of factitious Cushing's in which multiple investigations where done and a unilateral adrenelectomy was performed.

## Case Presentation

A 26 year-old woman with diagnosis of orofacial granulomatosis was referred for the evaluation of possible Cushing's syndrome. She reported a 15-kg weight gain and facial erythema. She had received prednisone for orofacial granulomatosis 2 years ago for 2 weeks prescribed by her dermatologist, but she denied any corticosteroid use thereafter. She denied any symptoms of hyperandrogenism or virilization such as acne, hirsutism, seborrhea, balding, or deepening of the voice. She had regular menstrual cycles and was not taking oral contraceptives. She had no history of diabetes or hypertension. Her family history included a brother who works as a nurse. Physical exam revealed a BMI of 22.4 kg/m^2^, with no facial plethora or skin striae. No hirsutism, acne, spotty pigmentation, or skin myxomas were noted. Thyroid examination was normal. The clinical suspicion for Cushing's syndrome was low. We ordered a low-dose dexamethasone suppression test and reschedule the patient for a 3-month follow-up. At 3 months follow-up she had developed new symptoms such as proximal muscle weakness, facial plethora, and reddish purple striae. Physical examination revealed Cushingoid features with moon face, supraclavicular fat pads and facial plethora. In addition, reddish purple striae >1 cm wide and proximal myopathy were noted.

**Figure 1:**
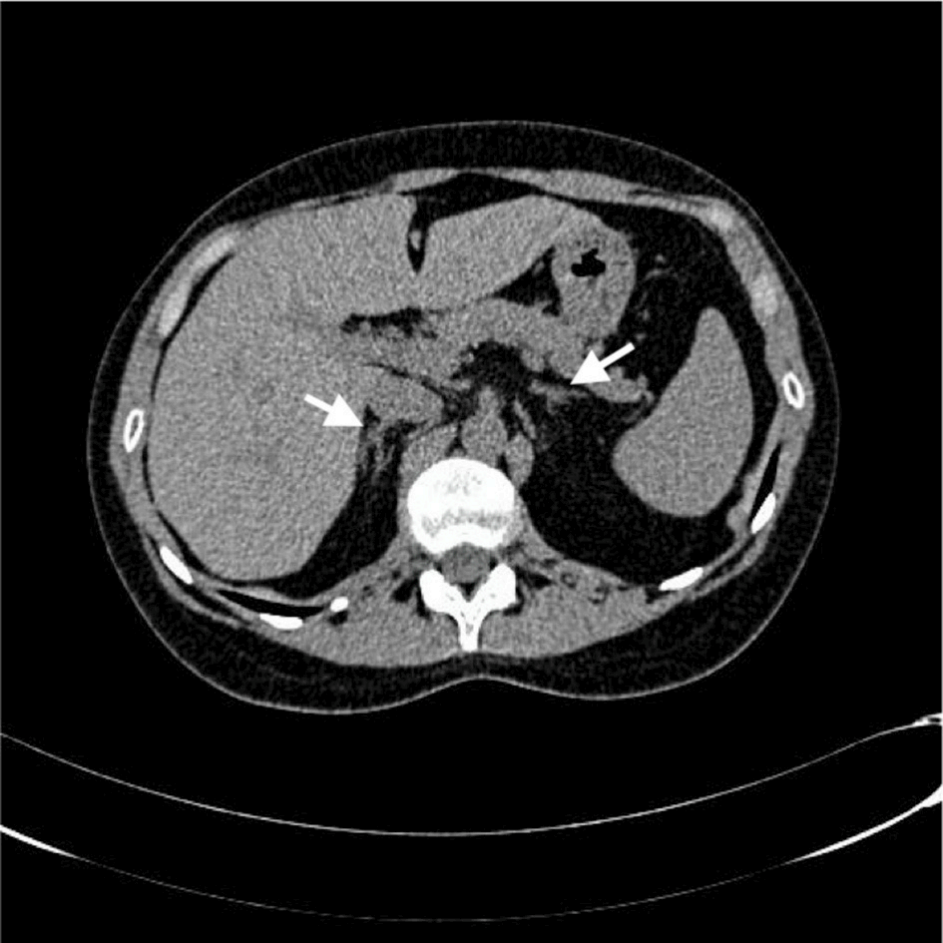
Computed tomography scan showing normal adrenal glands.

Work-up revealed an 8 a.m., serum cortisol of 6 μg/dl after 1 mg overnight dexamethasone suppression test (DST). Further work-up showed two consecutive elevated 24-h urinary free cortisol (UFC) (>510 μg/day and >485 μg/day, normal 20–90). The morning plasma adrenocorticotropic hormone (ACTH) was suppressed (<1 pg/ml, normal 7.2–63.3). These findings were consistent with an ACTH-independent Cushing's syndrome. She had normal complete blood count, LFT, KFT, and serum electrolytes. Lipid profile showed total cholesterol of 213 mg/dl, triglycerides of 90 mg/dl, HDL-C of 49 mg/dl, and LDL-C of 159 mg/dl. She was diagnosed with impaired fasting glucose (FPG of 104 and 109 mg/dl). Adrenal computed tomography (CT) scan showed no nodules or hyperplasia (Figure 1). With the suspicion of primary pigmented nodular adrenocortical disease a Liddle's test was ordered. During Liddle's test of 6 days (2 days of baseline collection, 2 days of 0.5 mg of dexamethasone orally every 6 h, and 2 days of 2 mg orally every 6 h) urinary cortisol increased from 71 to 413 μg/day. Basal urine free cortisol levels were within normal values (20–100 μg/day). It was interpreted as an intermittent variation of cortisol secretion. A primary pigmented nodular adrenocortical disease was suspected.

**Figure 2:**
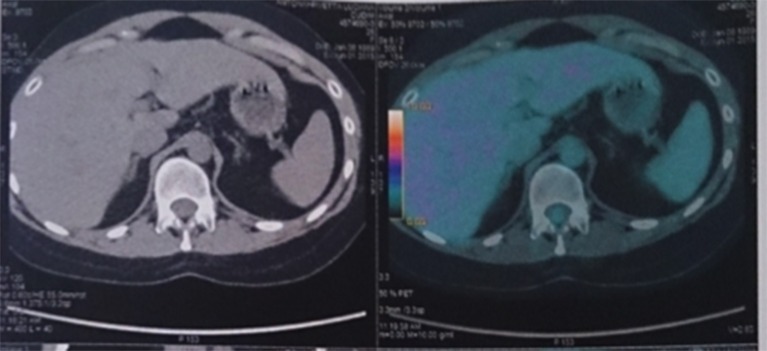
18F-FDG PET/CT scan showed no uptake in the adrenal glands.

Before any further work-up or treatment a synthetic glucocorticoid serum screening was ordered. The administrators denied it due to the fact that this test was not available in our hospital. A positron emission tomography (PET)-CT using 18F-flurodeoxyglucose (18F-FDG PET/CT) did not show any uptake in the adrenal glands (Figure 2). She had no laboratory findings consistent with Carney complex. No cardiac myxomas were noted on echocardiogram. Thyroid ultrasound was normal. With the diagnosis of probable primary pigmented nodular adrenocortical disease a unilateral right adrenelectomy was performed. We planned a two-staged laparoscopic adrenal resection, with subsequent contralateral adrenelectomy in a delayed surgery once we had pathological confirmation of PPNAD. However, histopathological examination revealed normal adrenal gland. We decided to perform a synthetic glucocorticoid serum screen outside our hospital, which was sent to Mayo Clinic lab. High levels of serum prednisone and prednisolone (2.9 and 12 mcg/dl, respectively-reference value <0.1 mcg/dl) performed by liquid chromatography-tandem mass spectrometry (LC-MS/MS) were found.

The psychiatrist at our hospital evaluated her. She was confronted with the evidence that her high steroid levels did not result from an endogenous production. She admitted taking steroids, prednisone 20 mg per day. She was diagnosed with a dependent and histrionic personality disorder. Further questioning revealed her boyfriend died around 2 years previously due to a car accident. She has a suppressed hypothalamic-pituitary-adrenal (HPA) axis (early morning cortisol <3 mcg/dl off glucocorticoid replacement for 24 h) treated with hydrocortisone 10 mg in the morning and 5 mg in the afternoon. She is currently under psychiatric care.

## Discussion

Despite the widespread use of glucocorticoids, there are few reported cases of factitious Cushing's syndrome. It represents <1% of patients with Cushing's syndrome. At the National Institute of Health Clinical Centre they found 6 cases of factitious Cushing's syndrome in 860 patients evaluated for hypercortisolism (5). In this review patient characteristics of factitious Cushing's syndrome included an occupation related to the medical field or close contact with a medical worker, a history of anxiety or depression and several surgeries for unrelated disorders. In this case the brother works as a nurse, without any other of the features described previously.

There are 23 cases reported in the literature. Eighty two percent were female, with a mean age of 37 years old (4–16). Almost half (43%) had a personal contact or occupation related to the medical field, and 57% history of psychiatric disorders. However, 4 cases presented with unsuppressed ACTH, which can mimic Cushing's disease (Table 1) (4–6). There are two fatal cases reported, due to pancreatitis and invasive aspergillosis (6, 15). To differentiate between endogenous and exogenous Cushing's syndrome may be difficult as physical findings are indistinguishable, and with similar psychiatric assessment (5). Differential diagnosis includes cyclic Cushing's, characterized by periodic fluctuations of cortisol secretion. Cycles of hypercortisolism alternate with phases of normal or low cortisol production, which can last days or years (17). It can be present in ACTH dependent or independent Cushing's syndrome. Approximately in 10% of cases an adrenal tumor is the underlying cause (17). In this case, although there was a paradoxical increase of cortisol with a 100% rise in 24 h UFC during the Liddle's test suggestive of PPNAD, basal urine free cortisol levels were within normal values. It was interpreted as an intermittent variation of cortisol secretion, maybe due to cyclic Cushing's. The pathophysiology of cyclic Cushing's is unknown. Proposed mechanisms of cyclic Cushing's include episodic hemorrhage and/or necrosis of the tumor resulting in temporary damage of secreting cells as well as synchronous growth and death of ACTH- or cortisol- secreting tumor cells (18–21).

**Table 1:** Clinical and laboratory data from patients with unsuppressed ACTH.

**References**	**Age (Years)**	**Gender**	**Symptoms**	**Plasma ACTH**	**Pituitary MRI**	**Adrenal CT scan**	**Medication ingested**
Cizza et al. (5)	44	Female	Depression, fatigue, bone pain	Normal	Questionable	Normal	Not know
Cizza et al. (5)	37	Female	Vertebral compression fractures	High	Microadenoma	Normal	Prednisolone/dexamethasone
Thynne et al. (4)	54	Female	Weight gain, fatigue, easy bruising, irritability, muscle weakness	Normal	Normal	Normal	Prednisolone
Minanni et al. (6)	26	Female	Depression, signs and symptoms of Cushing's syndrome	Normal	Microadenoma	Normal	Prednisone

There are few cases of pigmented nodular adrenocortical disease presenting as cyclic Cushing's (22, 23). Primary pigmented nodular adrenocortical disease is rare, responsible for <1% of Cushing's syndrome. The hipercortisolism is consequence of small-pigmented functioning nodules. Approximately 50% of cases are sporadic, the rest are familial ether isolated or as part of Carney complex (24). The paradoxical increase of UFC with dexamethasone during the Liddle's test is mediate by a glucocorticoid receptor overexpression in the nodules (25–27). Adrenal computed tomography is often interpreted as normal as the overall size of adrenal glands is not increased. However, the adrenal glands are occupied by small nodules that can be seen as string of beads on thin-section CT scan (24). Bandelin et al. reported a case in which PET-CT with 18F-flurodeoxyglucose showed uptake in both adrenal glands in a patient with PPNAD (28).

Laboratory features that suggest factitious Cushing's syndrome are erratic and fluctuating cortisol measurements as well as suppressed ACTH. In our case despite initial diagnosis of hypercortisolism with high UFC, during Liddle's test basal urine free cortisol levels were within normal values (71 μg/day).

Immunoassays measuring cortisol demonstrate cross-reactivity with synthetic corticosteroids and their metabolites. Exogenous glucocorticoid ingestion may yield high cortisol levels depending on the degree of cross-reactivity, the timing of ingestion, and the patient's endogenous cortisol secretion (4). Synthetic glucocorticoids can be detected by LC-MS/MS, which is the most important tool to rule out factitious Cushing's syndrome. Hospitalization with observation of patient and search of the room may be helpful to reach a diagnosis, more so in this case that synthetic glucocorticoid screen was not available (2, 29).

In cases with ACTH-independent Cushing's that presents as cyclic Cushing's and normal adrenals on CT scan glucocorticoid screen may be an extreme valuable tool to prevent unnecessary work-up and treatments.

Moreover, systems with electronic medical records may be more helpful to diagnose factitious disorder compared to those with paper charts (30). Analysis of previous medical records through centralized databases may give access not only to records in the doctor's hospital system but also to hospital networks. It may be helpful for doctor's to know all prescriptions that the patient received. However, in this case the prednisone prescription was documented in the paper chart and was spontaneously referred by the patient.

The pathogenesis of factitious disorder is unknown. It has been associated with psychosocial factors such as early losses due to death, abandonment or sickness (31). In this case, after psychiatric consultation death of her boyfriend was noted. In addition, it has been associated with neurocognitive impairment, such as dysfunction of the right hemisphere (32). In some cases it has been also associated with personality disorders such as borderline personality disorder (31). Once diagnosed, psychiatric care is warranted. Treatment of factitious disorder is extremely difficult. Confrontation with the diagnosis often leads to denial, accusations, leave, and seek medical care elsewhere or threatens of lawsuits (33). Psychotherapy is the standard treatment, comparable to that for personality disorder (31).

In addition, replacement doses of steroids are necessary to address the secondary adrenal insufficiency due to suppression of the HPA axis that remains after long-term corticosteroid use. Sudden withdrawal of glucocorticoids should be avoided, and instructions for emergencies such as illness or surgery must be addressed. These patients may have increase morbidity and mortality associated with Cushing's syndrome (34). In our case, her symptoms and signs of Cushing's syndrome have disappeared. She lost weight and she is still under psychiatric care.

## Concluding Remarks

In conclusion, factitious Cushing's syndrome represents a diagnostic challenge for physicians. It is an important diagnosis to consider in patients being evaluated for hypercortisolism. Discordant hormonal test results as well as normal findings on adrenal glands on CT scan should raise suspicion of this entity, and prompt measurement of synthetic corticosteroids using LC-MS/MS. In cases where synthetic glucocorticoid screen is not available and suspicion for factitious disorder is high hospitalization and waiting before sending the patient to surgery should be considered.

## Ethics Statement

The patient provided written informed consent for research participation as well as for the publication of indirectly identifiable data (age, gender, and medical history).

## Author Contributions

MP wrote the first draft of the manuscript. LR contributed to the writing of the manuscript. MP, LR, SD, LS, EB, VB, and VE made contributions to the acquisition of the clinical data. MP, LR, SD, LS, EB, VB, VE, DC, and MV agreed with manuscript results and conclusions. MP and VE made critical revisions and approved final version. All the authors revised and approved the final manuscript and agreed to be accountable for the content of the work.

### Conflict of Interest Statement

The authors declare that the research was conducted in the absence of any commercial or financial relationships that could be construed as a potential conflict of interest.
